# Analysis of the clinical manifestations and diagnostic process of the pleural effusion associated with constrictive pericarditis

**DOI:** 10.3389/fmed.2024.1419750

**Published:** 2024-11-15

**Authors:** Weifeng Wei, Panxiao Shen, Shaowei Liu, Naijian Li, Yunxiang Zeng, Lunchao Ma, Jinlin Wang

**Affiliations:** ^1^Pulmonary and Critical Care Medicine, State Key Laboratory of Respiratory Disease, National Center for Respiratory Medicine, Guangzhou Institute of Respiratory Health, The First Affiliated Hospital of Guangzhou Medical University, Guangzhou, China; ^2^Department of Cardiothoracic Surgery, The First Affiliated Hospital of Guangzhou Medical University, Guangzhou, China

**Keywords:** constrictive pericarditis, pleural effusion, clinical manifestations, diagnostic procedure, imaging features

## Abstract

**Objectives:**

The primary objective of this study is to analyze the clinical manifestations, diagnostic procedures, and outcomes of patients with pathologically confirmed constrictive pericarditis who presented with bilateral pleural effusions. We aim to outline a stepwise diagnostic approach that could assist clinicians in identifying CP in similar cases.

**Methods:**

In this study, we conducted a retrospective analysis of 19 cases of pathologically confirmed constrictive pericarditis. These patients were admitted to our hospital between January 2019 and December 2022 after pericardial stripping. The collected data included imaging findings, clinical manifestations, pleural effusion characteristics, postoperative pathology, and the diagnostic process.

**Results:**

In this study, the age of the 19 patients ranged from 25 to 74 years, with a median age of 59 years. All patients presented with bilateral pleural effusions, with or without pericardial lesions such as pericardial effusion or thickening. Pleural effusion biochemistry did not exhibit specific characteristics, and both etiology and pleural pathology were negative. Radiographic examinations, including cardiac ultrasound and chest CT, revealed signs of pericardial thickening and/or pericardial effusion or no abnormalities. Pericardial stripping was performed in all patients, and the postoperative pathology confirmed various degrees of thickened pericarditis, consistent with constrictive pericarditis. During a follow-up period of 6–18 months, most patients (17 out of 19) showed favorable recovery with no recurrence of bilateral pleural effusions.

**Conclusion:**

This study emphasizes the importance of pleural effusion as a clinical sign of constrictive pericarditis and highlights that a stepwise diagnostic approach, guided by clinical presentation and imaging, can enhance its recognition, particularly in cases with bilateral pleural effusions.

## Background

Pleural effusion is a common clinical problem with more than 50 recognized etiologies (1, 2). In the United States alone, approximately 1.5 million pleural effusions are diagnosed each year (3). Congestive heart failure (HF) and malignancy account for about one-third and one-tenth of these cases, respectively. While unilateral pleural effusions in adults are addressed by pleural disease guidelines proposed by the British Thoracic Society, less attention has been given to patients with bilateral pleural effusions (4). Unlike unilateral pleural effusions, the formation of bilateral pleural effusions can be attributed to a multitude of etiologies. Cardiac factors, particularly acute or chronic HF resulting from various heart diseases, are the most significant causes of unilateral pleural effusions (4). In addition to HF, pericardial diseases like constrictive pericarditis (CP) can also lead to the development of bilateral pleural effusions.

CP, although less commonly seen compared to bilateral pleural effusions, poses challenges in diagnosis (5). While idiopathic or post-viral pericarditis is the most common cause of CP, especially in the Western world, tuberculosis remains the major cause in developing regions (6–9). CP is characterized by impaired diastolic filling of the ventricles due to pericardial contraction, presenting as right heart failure with preserved right ventricular and left ventricular function (10). Pericardial thickening is a typical imaging presentation of CP, but initial diagnostic studies often yield nonspecific results, and the insidious onset and nonspecific symptoms contribute to diagnostic difficulties (5). Moreover, the clinical characteristics, examination procedures, and diagnostic approaches for CP are still not well-defined (6).

Notably, CP is strongly associated with pleural effusion, with up to 44% of CP cases presenting with pleural effusion (11), with left-sided presentations being more common (12). Right-sided or bilateral pleural effusions are more characteristic of HF. Although sporadic reports of bilateral pleural effusions secondary to CP exist, there is limited analysis of the clinical features, diagnostic procedures, and treatment of CP in such cases. Therefore, this study aimed to collect and analyze comprehensive clinical data from 19 patients with pathologically confirmed CP to better characterize the condition and outline diagnostic procedures. Importantly, a preliminary diagnostic procedure for CP was introduced.

## Methods

### Study population and clinical data collection

We conducted a retrospective analysis using data from 19 patients who presented with bilateral pleural effusions and were pathologically confirmed to have CP through pericardial stripping at the National Center for Respiratory Medicine in Guangzhou, P.R. China. The data collection period spanned from January 2019 to December 2022. The collected data encompassed various aspects, including demographic information such as age and gender, the duration of the disease (course), etiology, clinical manifestations, pleural effusions characteristics, imaging findings, pathology results, the degree of postoperative pericardial thickening, and follow-up information.

Inclusion Criteria: We selected patients who (1) presented with bilateral pleural effusions (2), were suspected of having pericardial disease based on clinical signs or initial imaging results, and (3) underwent pericardial stripping, with histopathological confirmation of constrictive pericarditis. Only patients with complete clinical, imaging, and pathological data were included in the study.

Exclusion Criteria: We excluded patients with (1) known etiologies for pleural effusions unrelated to cardiac or pericardial disease (e.g., malignancy, renal failure) (2), incomplete medical records or imaging data, and (3) conditions such as active infections or autoimmune diseases that could confound the clinical presentation.

Data Collection: Data were collected retrospectively from patient medical records, including clinical history, laboratory test results, imaging studies, and postoperative pathological findings. All imaging examinations, such as cardiac ultrasound and chest CT, were performed using standardized protocols at our hospital.

Imaging Techniques: Specifically, we used transthoracic echocardiography (TTE) to evaluate pericardial thickness and diastolic filling of the ventricles, and contrast-enhanced chest CT scans to assess pericardial thickening, pleural effusions, and the presence of pericardial calcification. In select cases, 18F-FDG PET/CT was employed to evaluate metabolic activity in the pericardium.

### Statistical methods

Continuous variables were shown as either mean (standard deviation, S.D.) or median (interquartile range, IQR) according to data distributions. Categorical variables were displayed as frequencies (percentages).

## Results

### Demographic characteristics

Patient-related clinical information, including demographic characteristics, clinical findings, and laboratory test results, was collected and documented in Table 1. More detail information was shown in Supplementary Table S1. The age of the participants ranged from 25 to 74 years, with a median age of 59 years. Among the patients, 17 were male, and 2 were female. Fifteen cases were aged 50 years or older, and the duration of the disease varied from 20 days to 24 months, with a median course of 4 months.

**Table 1:** Clinical information of 19 patients including: demographic data; serological examination; pleural effusion examination and clinical symptoms and signs.

*N*	Age y/(gender)	Serum levels							Pleural effusion levels					
		WBC (×10^9^/L)	N* (×10^9^/L)	ESR (mm/h)	NT-pro BNP (pg/mL)	TP (g/L)	LDH (U/L)	TB index	TP (g/L)	LDH (U/L)	ADA (U/mL)	Property	CEA (ng/mL)	NT-proBNP (pg/mL)
1	72/M	2.40	1.6	29	546.2	60.6	192.4	*N*	25.2	92.0	4.0	A	0.47	627.4
2	71/M	3.80	2.0	64	470.5	60.1	211.9	*N*	21.4	97.2	0.6	A	1.39	726.1
3	61/M	5.60	3.5	57	592.4	69.0	220.2	*N*	32.6	100.8	8.1	A	0.95	733.8
4	56/M	5.40	3.8	66	807.1	69.8	241.2	*N*	43.4	172.0	10.3	B	0.80	941.4
5	50/M	5.18	3.8	50	318.8	63.4	407.0	*N*	45.2	1840.9	8.6	B	0.82	521.9
6	65/F	3.30	2.2	6	1213.0	54.8	286.5	*N*	29.7	132.0	0.2	B	0.67	1365.0
7	63/M	8.20	5.2	60	550.0	59.2	250.0	*N*	28.8	267.8	9.2	B	0.75	320.0
8	57/M	4.90	3.3	28	386.6	63.6	269.7	*N*	16.4	107.4	9.0	A	0.87	724.0
9	69/M	6.20	3.3	33	480.2	72.9	251.5	*N*	35.4	128.5	3.6	B	1.00	593.8
10	74/M	3.10	2.3	12	3345.0	56.7	239.0	*N*	24.6	122.0	17.8	A	1.76	3162.0
11	25/M	6.58	4.4	29	331.4	70.0	287.0	*N*	29.4	169.0	0.9	B	0.46	413.0
12	59/M	5.20	2.5	26	77.9	66.1	215.2	*N*	21.3	114.8	3.7	A	0.85	85.9
13	33/M	9.40	6.2	69	2167.0	56.4	239.5	*N*	17.9	103.8	0.9	A	0.44	2264.0
14	36/M	9.70	8.2	103	348.0	69.7	116.0	*N*	40.1	695.5	21.8	B	0.85	347.0
15	72/M	5.10	3.9	20	2239.0	66.4	250.9	*N*	30.1	280.0	19.1	B	1.35	2203.3
16	50/M	6.60	3.8	110	780.0	62.5	220.0	*N*	28.2	134.0	8.6	B	0.47	935.0
17	47/M	4.80	2.8	40	564.0	70.5	178.3	*N*	29.5	255.5	8.5	B	0.91	524.0
18	65/M	3.64	2.5	48	610	61.2	366.9	*N*	22.9	161.8	19.4	A	0.99	900
19	65/F	4.20	3.2	78	559	60.7	275.2	*N*	35.8	165.5	13.7	B	1.80	595

WBC white blood cell, N* Neutrophil, ESR erythrocyte sedimentation rate, NT-pro BNP N-Terminal Pro-Brain Natriuretic Peptide, TP total protein, CEA carcinoembryonic antigen, LDH lactate dehydrogenase, ADA adenosine deaminase, TB index, Tuberculosis infection-related index, including sputum smears, T-spot, Tuberculosis-DNA (TB-DNA) and bacillus tuberculosis culture, A, transudate; B, exudate; N, negative.

### Clinical findings

All 19 patients presented with bilateral pleural effusions and pericardial effusions. The most common symptoms reported were dyspnea (17/19, 89.5%) and cough (9/19, 47.4%). Less common symptoms included chest pain (3/19, 15.8%), fever (2/19, 10.5%), and abdominal distension (2/19, 10.5%). Only one of these cases had a previous history of tuberculosis. Clinical signs of CP included pleural effusions (19/19, 100%) and signs of systemic venous congestion, such as edema of the lower extremities (9/19, 47.4%), ascites (3/19, 15.8%), jugular venous distention (2/19, 10.5%), hepatojugular reflux (1/19, 5.3%), and scrotal edema (1/19, 5.3%) (refer to Table 1).

### Laboratory tests for blood and pleural effusions

Blood analyses were performed on all patients (Table 1). Among the cases, three (Case 10, 13, and 15) exhibited significantly elevated levels of N-terminal pro-B-type natriuretic peptide, all exceeding 2000 pg/mL (mean 2583.6 pg/mL). Interestingly, N-terminal pro-B-type natriuretic peptide levels in the pleural effusions were also elevated (mean 2543.1 pg/mL). No specific abnormalities were observed in the blood tests, including white blood cell count, neutrophil count, liver function, kidney function, erythrocyte sedimentation rate, carcinoembryonic antigen, and lactate dehydrogenase. Furthermore, tests for tuberculosis infection, including sputum smears, T-SPOT.TB test, tuberculosis-DNA, and Bacillus tuberculosis culture, all yielded negative results.

We examined pleural effusions specimens from all 19 patients, identifying 11 as exudates and 8 as transudates based on Light’s criteria (pleural effusions/serum protein ratio > 0.5, pleural effusions/serum lactate dehydrogenase (LDH) ratio > 0.6, pleural effusions/upper limit of normal serum LDH ratio > 2/3). The mean concentrations of adenosine deaminase, carcinoembryonic antigen, and protein in the pleural effusions were 8.8 U/L (range: 0.2–21.8 U/L), 0.92 mg/mL (range: 0.4–1.8 mg/mL), and 29.3 g/dL (range: 16.4–45.2 g/dL), respectively. The median concentrations of LDH and N-terminal pro-B-type natriuretic peptide in the pleural effusions were 134.0 U/L (range: 92.0–1840.9 U/L) and 724.0 pg/mL (range: 85.9–3129.0 pg/mL), respectively. Similar to the previous findings, tests for tuberculosis infection, including tuberculosis smear, T-SPOT. TB test, tuberculosis-DNA, and bacillus tuberculosis culture, all yielded negative results.

### Radiologic features

All 19 patients exhibited bilateral pleural effusions with pericardial lesions observed on imaging, including pericardial effusion and/or pericardial thickening. Additionally, 13 cases were accompanied by peritoneal effusion. Cardiac ultrasound revealed pericardial thickening in 9 cases, restricted diastolic filling in both the right and left ventricular walls in 7 cases, and ventricular septal bounce-like motion in 5 cases. Pericardial effusion was observed in 15 cases, left atrial enlargement in 3 cases, and no abnormalities in 2 cases. All patients had normal ejection fraction values (with a mean Left Ventricular Ejection Fraction value of 66), and no significant changes were observed after pericardial dissection. Abdominal ultrasound indicated liver congestion in 4 cases and widening of the inferior vena cava in 3 cases. Chest CT scans showed bilateral pleural effusions in all 19 patients, pericardial thickening in 17 cases, and pericardial effusion in 14 cases. Among the patients, significant pericardial thickening was observed in 89.5% (17/19) with a mean thickness of 8.4 mm (range: 5.5–17.3 mm) in the mediastinal window of the chest CT (refer to Table 2).

**Table 2:** Summary of the patient’s imaging characteristics.

Imaging characteristics	Frequency^a^ (*N* = 19)
Ultrasonography	19 (100)
Bilateral pleural effusion	19 (100)
Pyoperitoneum	13 (68)
Pericardial effusion	15 (78)
Ultrasound of the heart	19 (100)
No abnormalities	2 (11)
Pericardial thickening	9 (47)
Restricted diastolic filling	7 (37)
Ventricular septal bounce-like motion	5 (26)
Pericardial effusion	15 (78)
Atrial enlargement	3 (16)
Ultrasound of the abdomen	19 (100)
Hepatic congestion	4 (21)
Widening of the inferior vena cava	3 (15)
Chest CT	19 (100)
Bilateral pleural effusion	19 (100)
Pericardial effusion	14 (73)
Pericardial thickening	17 (89)
The mean thickness of pericardium	8.4 mm

^a^Values provided as No. (%).

**Figure 1:**
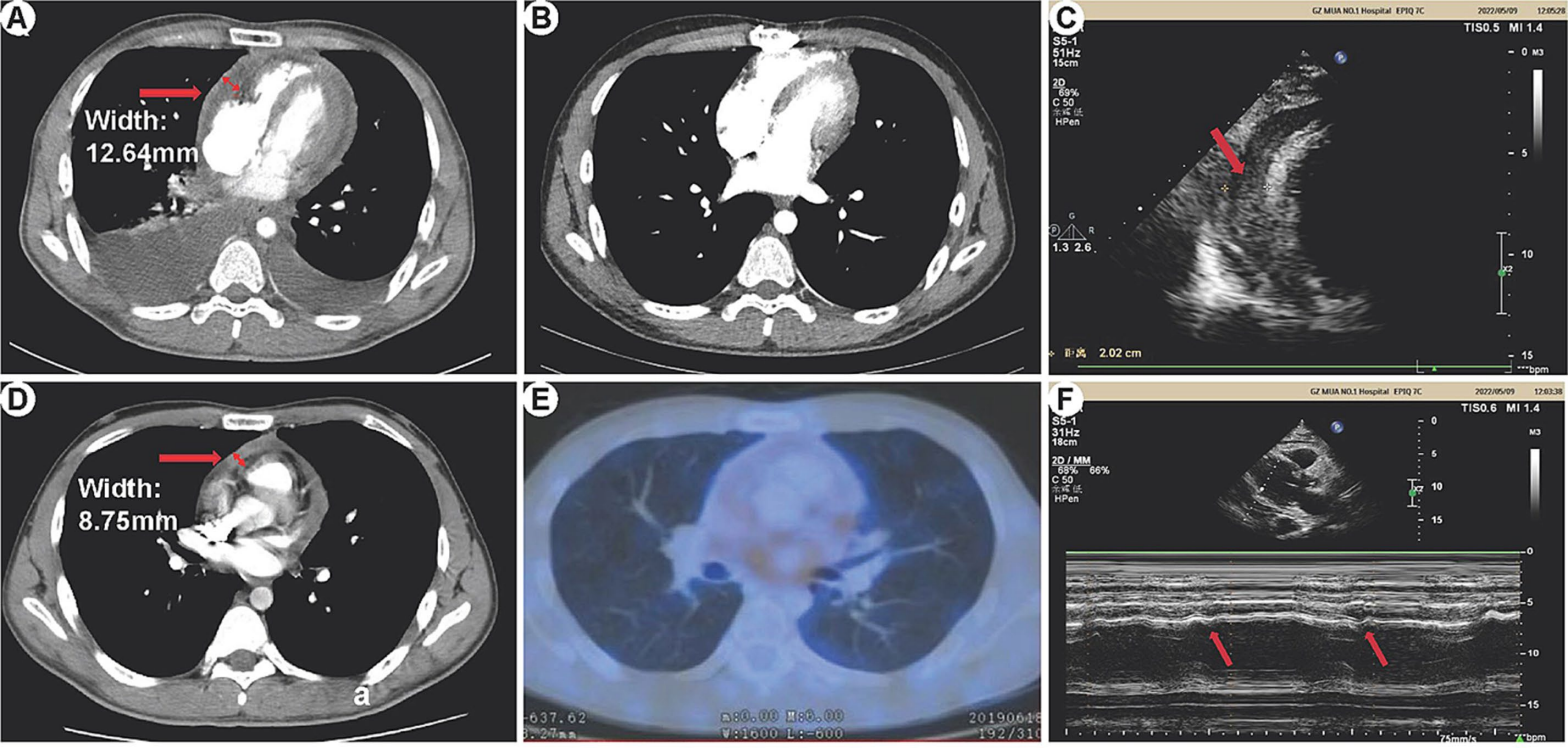
HRCT, 18F-FDG PET/CT, and echocardiography manifestations of constrictive pericarditis. **(A)** Preoperative CT: Bilateral pleural effusion is visible, more prominent on the right side. The pericardium appears significantly thickened, measuring 12.64 mm at its thickest part. **(B)** Postoperative CT: No pleural effusion is observed in either chest cavity. There is no evidence of pericardial thickening or pericardial effusion. **(C)** Echocardiography: The ventricular septum exhibits bouncing motion (indicated by arrows), characterized by a short backward motion in early diastole. **(D,E)** 18F-FDG PET/CT: Pericardial thickening is observed with unevenly increased glucose metabolism. The maximum thickness of the pericardium measures 8.75 mm. **(F)** Echocardiography: The pericardium displays inhomogeneous high-weak echogenic changes, indicative of pericardial thickening (arrows).

In this study, 3 patients underwent 18F-FDG PET/CT examination, revealing pericardial thickening in 2 cases. These cases exhibited a significant metabolic increase with maximum standardized uptake values of 13.7 and 2.9, respectively. One case showed a small amount of pericardial effusion, while no hypermetabolic lesions were observed. We presented the imaging results of one patient (patient 11) as an example (refer to Figure 1): Preoperative CT scans demonstrated bilateral pleural effusions and significant pericardial thickening (Figure 1A). The preoperative 18F-FDG PET/CT also revealed pericardial thickening, showing an uneven increase in glucose metabolism within the thickened pericardium (Figures 1D,E). Echocardiography depicted ventricular septum exhibiting bouncing motion and an inhomogeneous high faint echogenic change in the pericardium, suggesting pericardial thickening (Figures 1C,F). Post-pericardial stripping CT scans did not show PE or significant pericardial thickening (Figure 1B).

### Postoperative pathology

**Figure 2:**
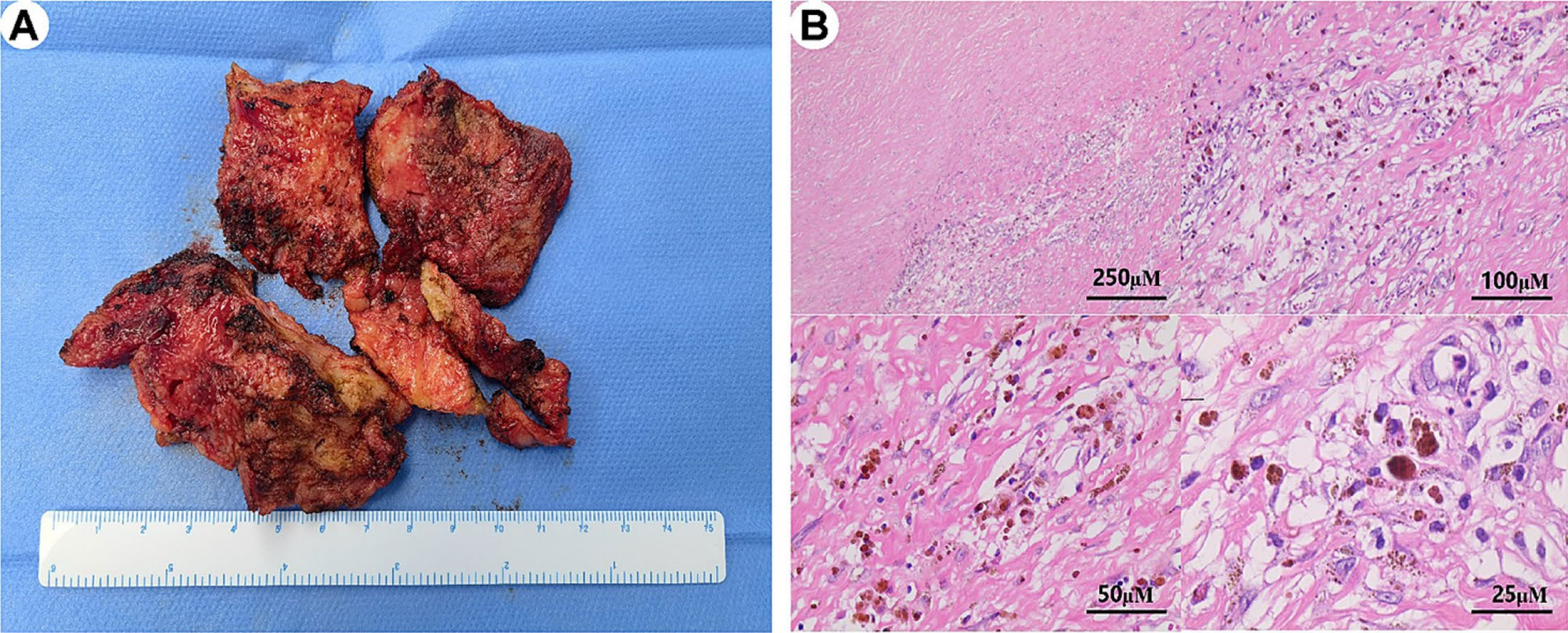
Morphological characteristics of postoperative paraffin sections in constrictive pericarditis. **(A)** The stripped pericardial tissue is shown in several parts. **(B)** The pathological analysis of the pericardium reveals extensive fibrous tissue hyperplasia with vitreous degeneration, scattered lymphocyte and histiocyte infiltration, deposition of iron-containing heme cells, and vascular hyperplasia.

All patients underwent pericardial stripping, the detached pericardial tissue of one patient (patient 11) is shown in Figure 2A. All patients’ stripped pericardium were subjected to pathological examination, which confirmed two types of postoperative pathology: (1) 14 cases presented with typical manifestations of chronic CP (non-tuberculous type), characterized by significant fibrous tissue hyperplasia with collagenization, fibrinous exudation (in some cases), scattered lymphocyte infiltration, histiocyte infiltration, and deposits of iron-containing heme cells (Figure 2B); (2) A total of 5 cases presented with manifestations of tuberculous pericarditis, characterized by granulomatous changes or caseous necrosis, with or without acid-fast staining (+).

## Discussion

CP is characterized by the heart being surrounded by a dense, thickened, fibrotic, or calcified pericardium, which restricts diastolic filling of the ventricles and leads to various circulatory disturbances (13, 14). Most patients with CP experience symptoms of chronic heart failure, and the presence of pleural effusion is a relatively common clinical manifestation, which can be exudative or transudative in nature (11, 15, 16). However, Hooper et al. demonstrated that CP is considered a rare cause of exudative pleural effusions (17). Due to its rarity, accurate diagnosis poses significant challenges for clinicians.

Although there have been numerous studies on CP, only a few cases of pleural effusions caused by CP have been reported. In our study, we analyzed the clinical features of 19 CP patients who presented with bilateral pleural effusions as the initial manifestation, aiming to elucidate the diagnostic procedures. The most common symptoms observed in CP were dyspnea and cough, and the signs predominantly indicated systemic venous congestion, which aligned with the clinical presentation of the patients in our study (18). However, these clinical manifestations are not exclusive to CP and can be observed in patients with other conditions. Previous studies indicated that tuberculous pericarditis was considered the primary etiology of constrictive pericarditis in developing countries, including China. Nevertheless, confirmed tuberculous pericarditis accounted for only 26% of cases in our study. All tuberculosis tests, including sputum culture, sputum smear, and tuberculosis-DNA, yielded negative results in our study population. Consequently, some patients were initially misdiagnosed due to the absence of specific clinical presentations and laboratory tests.

A stepwise approach has been emphasized in the investigation of pleural effusions (19). In our study, we performed thoracentesis on all patients to determine the nature of the pleural effusions. According to Light’s criteria, 10 patients had exudative pleural effusions, while 9 patients had transudative pleural effusions. The levels of carcinoembryonic antigen, adenosine deaminase, and LDH in the pleural effusions were nonspecific for these 19 patients. As common pulmonary causes of pleural effusions, the diagnoses of malignancy, inflammation, and tuberculosis needed to be clarified (4). However, inflammation was ruled out as none of the infection indicators were elevated. In cases where the cause of exudative pleural effusions remained inconclusive, a pleural biopsy was deemed necessary for a definitive diagnosis, in order to exclude malignancy as well as tuberculosis (4, 20). Therefore, pleural biopsies were performed on patients presenting with exudative pleural effusions. No evidence of tuberculosis or malignancy was found in the patients who underwent pleural biopsies.

The diagnosis of CP relies on imaging features to differentiate it from other diseases. Echocardiography, which provides a comprehensive morphofunctional assessment of the heart, is currently the imaging method of choice for the diagnostic evaluation of CP (6, 21). CT is commonly used as a non-invasive test to assess pericardial thickness (9). Typical imaging features include pericardial thickening or pericardial effusion on cardiac CT, as well as restricted diastolic filling of the ventricular wall and respiration-related ventricular septal shift on cardiac ultrasound. However, it is important to note that not all patients may exhibit pericardial lesions on CT, and there is a possibility of missed diagnoses on echocardiograms due to operator subjectivity. Therefore, it is advisable to combine various cardiac imaging tests, including cardiac CT, MR, and 18F-FDG PET/CT, to enhance the accuracy of the diagnosis. In two cases, although CT imaging did not reveal pericardial thickening, the clinical presentation and echocardiographic findings were strongly suggestive of CP. Both patients exhibited characteristic echocardiographic features such as ventricular septal bounce and restricted diastolic filling, along with significant clinical symptoms such as dyspnea and signs of right heart failure. Given these clinical and echocardiographic findings, pericardial stripping was deemed necessary.

**Figure 3:**
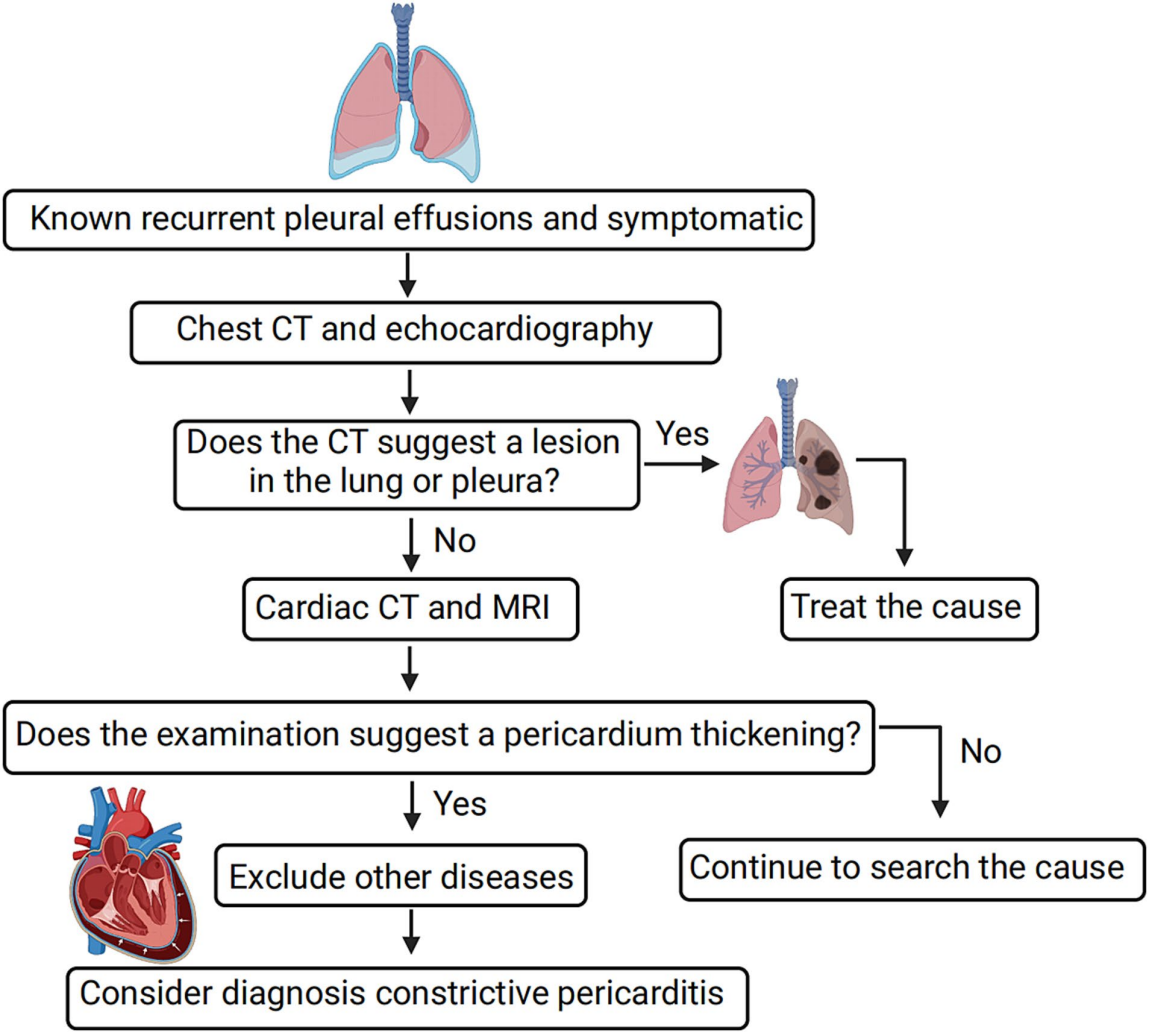
Simple diagnostic procedure for constrictive pericarditis leading to bilateral pleural effusion. For diagnosing constrictive pericarditis, comprehensive imaging methods like cardiac CT, MR, and 18F-FDG PET/CT are essential, with a final diagnosis reached through the exclusion of other causes. Positive pericardial thickness indicates the detection of pericardial thickening or other suggestive features of CP on imaging modalities such as CT or echocardiography.

Based on our experience in managing 19 patients in this study, we have developed a preliminary diagnostic procedure for CP to enhance its detection (refer to Figure 3). The procedure involves several steps. Firstly, a routine CT and echocardiography examination is recommended be performed to assess the presence of lung and pleural lesions. If any lesions are identified, further examination, such as biopsy of the lesions, is necessary for a more detailed evaluation. Secondly, if typical manifestations of CP are indicated during the evaluation, additional cardiac imaging tests, such as cardiac CT, MR, and 18F-FDG PET/CT, are recommended be performed. These tests help identify cardiac lesions and contribute to a comprehensive diagnosis. Finally, the diagnosis of CP is established by excluding other potential causes through a systematic evaluation of clinical findings, imaging results, and laboratory tests. By following this diagnostic procedure, clinicians can enhance the accuracy of CP diagnosis and minimize the likelihood of misdiagnosis.

Despite the valuable insights gained from our study, it is important to acknowledge its limitations. Firstly, only a proportion of patients underwent routine 18F-FDG PET/CT examinations, and none of the patients had routine cardiac MR examinations. This may have influenced the comprehensiveness of our findings and their applicability to the broader population. Furthermore, it is crucial to note that our study was conducted as a single-center retrospective analysis, and the sample size was relatively small. Consequently, the generalizability of our results may be limited. To ensure the robustness and applicability of our findings, future research should encompass larger sample sizes and involve multiple centers, employing a prospective study design.

## Conclusion

Patients presenting with bilateral pleural effusions and pericardial lesions should raise suspicion for CP. Cardiac ultrasound and CT could aid in its diagnosis, and a comprehensive evaluation of multiple imaging examinations could improve the accuracy of diagnosing and managing CP. Pericardial dissection serves as a confirmatory diagnostic method and an effective treatment approach.

## Data Availability

The original contributions presented in the study are included in the article/Supplementary material, further inquiries can be directed to the corresponding author.
